# Readiness to implement contingency management to promote PrEP initiation and adherence among people who inject drugs: results from a multi-site implementation survey

**DOI:** 10.1186/s13722-024-00503-4

**Published:** 2024-12-23

**Authors:** Eleanor Pickering, Adam Viera, Minhee L. Sung, Daniel Davidson, Genie Bailey, Marianne Buchelli, Mark Jenkins, Jennifer Kolakowski, Leah Maier, E. Jennifer Edelman, Carla J. Rash

**Affiliations:** 1 Department of Social and Behavioral Sciences, Yale University School of Public Health, New Haven, CT 06510 USA; 2 Yale University School of Nursing, 06477 Orange, CT USA; 3 Collaborative Center to Advance Health Services, University of Missouri Kansas City School of Nursing and Health Studies, Kansas City, MO 64108 USA; 4 Center for Interdisciplinary Research on AIDS, Yale University School of Public Health, New Haven, CT 06510 USA; 5 VA Connecticut Healthcare System, West Haven, CT 06516 USA; 6 Yale Program in Addiction Medicine, Yale School of Medicine, New Haven, CT USA; 7 Stanley Street Treatment and Resources (SSTAR) Inc, Fall River, MA 02720 USA; 8 Connecticut Department of Public Health, 06134 Hartford, CT USA; 9 Connecticut Harm Reduction Alliance, 06106 Hartford, CT USA; 10 Recovery Network of Programs, Inc, Bridgeport, CT 06604 USA; 11 Apex Community Care, 06810 Danbury, CT USA; 12 Department of Internal Medicine, Yale School of Medicine, New Haven, CT 06510 USA; 13 University of Connecticut School of Medicine, Farmington, CT 06032 USA

**Keywords:** Contingency management, Pre-exposure prophylaxis, HIV prevention, Injection drug use, Implementation science

## Abstract

**Background:**

Contingency management (CM), an incentive-based intervention to encourage target behaviors, effectively promotes medication adherence. However, efforts to extend CM to HIV pre-exposure prophylaxis (PrEP) have been lacking. As part of a randomized clinical trial to promote HIV Prevention among people who inject drugs (PWID), we examined the readiness of staff in community-based organizations serving PWID to implement CM for PrEP uptake and adherence in this population.

**Methods:**

From April to August 2022, we conducted a survey of staff from four community-based organizations providing HIV testing, harm reduction, and outreach services in the northeastern United States. We assessed knowledge and attitudes regarding PrEP for PWID on five-point Likert scales (e.g., Poor to Excellent, Not at all to Extremely). Using a modified version of the Contingency Management Beliefs Questionnaire, we assessed the degree to which attitudes about CM for HIV prevention influenced interest in its adoption on a scale from “1-No influence at all” to “5-Very strong influence”. We explored endorsement patterns, along with average values of individual items and subscale scores.

**Results:**

Among 271 staff invitations, 123 (45.4%) responded. The majority (88.6%) of respondents reported prior PrEP awareness, with a mean self-rated knowledge of 2.98 out of 5 (SD = 1.1). Attitudes towards PrEP, including its relevance to and importance for clients (both means = 4.3), efficacy (mean = 4.5), and safety (mean = 4.2), were positive. Items related to practicality and confidence in providing PrEP-related care had relatively lower ratings. Respondents endorsed influential generalized (mean = 2.1) and training-related (mean = 2.5) CM implementation barriers less frequently than positive attitudes towards CM (mean = 3.6). Staff favored adding CM to existing services (mean = 3.8), and highly endorsed it as “useful for targeting HIV prevention with PrEP” (mean = 3.7).

**Conclusions:**

Respondents generally supported the use of CM to promote HIV prevention among PWID and favored adding it to their existing services. Though respondents understood the value of both PrEP and CM to support HIV prevention activities, findings corroborate research citing relative lack of knowledge and confidence regarding PrEP management among clients, potentially detracting from implementation readiness.

**Trial Registration Number:**

NCT04738825.

## Background

Pre-exposure prophylaxis for HIV prevention (PrEP) is recommended for people who inject drugs (PWID), who remain at elevated risk for contracting HIV as highlighted by multiple HIV outbreaks occurring in this group in the past decade [1, 2]. Despite moderate-to-high interest in PrEP among this group [3], engagement in PrEP remains extremely low for a variety of individual, institutional, and structural reasons [4–7]. Integration of PrEP linkage interventions in a variety of substance use service and treatment settings has been identified as a key strategy for increasing PrEP implementation among PWID [8].

Contingency management (CM), the use of incentives to promote verifiable behavior change, has been utilized across the HIV care continuum [9], with demonstrated success in improving HIV-related healthcare visit attendance, increasing adherence to HIV antiretroviral therapy, and maintaining suppressed HIV viral load [10–14]. Among people who use drugs, CM decreases substance use [15–17], and has been used effectively to promote treatment of opioid use disorder and infectious disease (including HIV) [18, 19], but has low rates of implementation outside of research settings [20, 21]. However, CM has not, to our knowledge, been applied to the uptake of and sustained adherence to PrEP among PWID, which remains an area of ongoing research [22, 23]. Progress towards achieving PrEP adherence can be verified in several ways, including documentation of an appointment with a clinician; evidence of laboratory testing needed prior to starting PrEP; evidence of medication fill; evidence of tenofovir metabolites in urine; and documentation of receipt of injectable PrEP.

Though issues of low PrEP initiation and adherence among PWID [24–28] may be directly addressable with CM, attitudes of clinical and non-clinical staff toward the use of CM for this purpose are poorly understood and represent potential implementation barriers. Consistent with a hybrid type 1 effectiveness-implementation approach [29], we sought to identify implementation barriers and facilitators of promoting PrEP for PWID by assessing baseline PrEP-related knowledge and attitudes among both clinical and non-clinical staff, in conjunction with their beliefs about CM for this population.

## Methods

From April to August 2022, we conducted a confidential survey of staff and clinicians from four community-based organizations participating in a randomized clinical trial of a stepped care intervention including CM and navigation services (“PrEP adherence and support services”) to promote HIV prevention with PrEP among PWID. This survey was conducted during the first year after launch of the parent randomized clinical trial (details have been published previously) [30].

Drawn from validated measures and previous surveys [31, 32], items for this survey were developed with interdisciplinary input and pilot tested prior to implementation.

### Participants and setting

Sites were intentionally selected because of their varying experiences participating in research, missions, and diversity in services. All four of the participating organizations provide on-site HIV testing, harm reduction, and outreach services; three of the organizations offer onsite medications to treat OUD; and two of the organizations house on-site PrEP care [30].

Each site-based Principal Investigator or designee identified a list of eligible staff – including administrative staff, frontline service providers, and leadership – to generate the final sample. To be considered eligible for the study, participants needed to be: (1) currently employed at one of the participating sites and engaged in directly or supervising service delivery; and (2) willing to complete the survey. For this survey, participants were given basic definitions of CM (that it “used rewards or prizes to incentivize behavior change”) and activity contracting (referring to the practice of working with clients to determine targeted behavior and respective source of verification that will be completed to earn CM rewards). This survey was administered as a baseline assessment of attitudes and beliefs, and most staff had not yet received extensive training on CM or PrEP as part of the parent trial [30].

### Measures

#### Participant characteristics

We assessed both sociodemographic (e.g., gender, race, HIV status) and professional (e.g., job type, experience at organization, HIV certification) characteristics. For potentially sensitive items such as HIV status, we included a ‘prefer not to answer’ option. Both organizational and total experience items were collapsed to ‘0–2 years’, ‘2–5 years’, and ‘5 + years’, to better understand employment status. Respondent sociodemographic and professional characteristics are presented in Tables 1 and 2, respectively.

**Table 1:** Respondent sociodemographic characteristics (*n* = 123)

Characteristic	*N* (%) or Med (IQR)
Age	45.0 (33.0, 56.0)
Gender	
Female Male Non-Binary	93 (75.6%) 28 (22.8%) 2 (1.6%)
Race	
Black or African American White Other	28 (22.8%) 76 (61.8%) 19 (15.5%)
Ethnicity	
Hispanic/Latinx Not Hispanic/Latinx	20 (16.3%) 103 (83.7%)
Highest Level of Education	
More than bachelor’s degree Bachelor’s degree More than high school High school	39 (31.7%) 32 (26.0%) 42 (35.2%) 10 (8.1%)
Identifies as a person in recovery^*^	26 (21.1%)
Identifies as having or at risk for HIV^ǂ^	10 (8.1%)

^*^9 participants preferred not to answer

^ǂ^3 participants preferred not to answer

**Table 2:** Respondent PrEP knowledge and professional characteristics (*n* = 123)

Characteristic	*N* (%) or Mean (SD) or Med [IQR]
Prior awareness of PrEP	109 (88.6%)
Self-rated PrEP knowledge (*n* = 109) Excellent Very Good Good Fair Poor	2.98 (1.1) 10 (9.2%) 26 (23.9%) 33 (30.3%) 32 (29.4%) 8 (7.3%)
Primary Role	
Administrator Medical prescriber Nurse Mental health provider Misc. direct service provider Other	8 (6.5%) 15 (12.2%) 30 (24.4%) 31 (25.2%) 24 (19.5%) 15 (12.2%)
Time in current profession	
More than five years Two to five years Less than two years	51 (41.5%) 41 (33.3%) 31 (25.2%)
Time at organization	
More than five years Two to five years Less than two years	62 (50.4%) 36 (29.3%) 25 (20.3%)
Current caseload (# of clients)	40 [10.0, 95.0]

#### PrEP knowledge and attitudes

PrEP knowledge was measured by respondent report of ever having heard of PrEP—a “yes/no” question—and self-rated knowledge of PrEP on a five-point Likert scale from “1 – Poor” to “5 – Excellent” (Table 2). Staff opinions about PrEP’s overall effectiveness, safety, and relevance; the appropriateness and practicality of PrEP-related care in respondents’ roles and at their organization; and their confidence about PrEP adoption and management with their specific clients were also captured in five-point Likert responses.

#### Contingency Management beliefs questionnaire

We collected information about respondent attitudes towards CM using an adapted version of the 32-item Contingency Management Beliefs Questionnaire (CMBQ). The CMBQ consists of three subscales focused on generalized barriers, training-related barriers, and support for CM [31]. We added 15 items related to its use to promote PrEP and medications for opioid use disorder. All items are statements with a 5-item Likert scale for response options, assessing the degree of influence each item had on the interest (or lack of interest) in implementing CM interventions for HIV prevention. The responses ranged from “No influence at all” (rating = 1) to “Very strong influence” (rating = 5). All items were reviewed by the interdisciplinary research team for clarity and relevance before being submitted to the IRB for approval. The data collection instrument can be found as supplementary material in the protocol paper for the larger study [30].

#### Data collection

The web-based survey was sent to the emails of identified staff and administered via REDCap (Research Electronic Data Capture) [33, 34] and supplemented by paper versions as preferred. The survey was administered after a single staff person from each site had all been trained on the intervention and its components (i.e., CM, PrEP navigation) and enrollment in the parent randomized control trial had commenced. Staff who did not complete the survey after two weeks were sent a reminder every two weeks until they completed the survey or the collection period ended.

#### Data analysis

We used R version 4.3.1 to generate descriptive statistics for all demographic and professional background variables. We calculated the average rating for each item and subscale of the modified CMBQ as well as all PrEP practice and attitude-related assessments. No analyses of association were conducted as the sample size did not offer sufficient power.

## Results

### Participant characteristics

Among 271 invitations, we received 123 (45.4%) complete responses. Respondents primarily identified as female (75.6%), White (61.8%), and not Hispanic or Latinx (83.7%) (Table 1). Over one in five identified as a person in recovery (21.1%) while just under one in ten identified as having or at risk for HIV (8.1%). In terms of their professional role and background, respondents most commonly identified as mental health providers (25.2%), nurses (24.4%), or some other direct service provider (19.5%). Most respondents had been in their current profession (74.8%) and at their respective organization (79.7%) for over two years (Table 2).

### PrEP knowledge and attitudes

One hundred nine (88.6%) respondents indicated having awareness of PrEP before taking the survey. Of the respondents who had prior PrEP awareness, self-rated knowledge ranged from 1.0 to 5.0, with an average rating of 2.98. When asked directly about their opinion, staff endorsed PrEP as relevant and important for clients (mean scores = 4.3), efficacious (mean score = 4.5), and safe (mean score = 4.2). Based on a five-point Likert scale from “Strongly Disagree – 1” to “Strongly Agree – 5”, respondents generally agreed that clients had access to PrEP within their organization (mean score = 4.5) and disagreed that PrEP-related care was impractical due to competing priorities (mean score = 2.7) or not within the confines of their role (mean score = 2.6).

While respondents reported confidence in knowing where to refer their clients for PrEP (mean score = 4.0), they were less confident that they knew enough about best practices (mean score = 2.8) and having the skills and knowledge necessary to assist clients in taking PrEP (mean score = 2.7). Findings suggested concerns with client capacity as well, with lower confidence in client motivation (mean score = 2.5) and ability to adhere to and cover the cost of PrEP (mean scores = 2.8 and 2.3, respectively). General response breakdowns are presented in Fig. 1a, 1b, 1c and item-by-item response breakdowns as well as means are presented in Table 3.

**Table 3:** Respondent PrEP attitude breakdown

	N (%) (*n* = 123)					Mean (SD)
PrEP Opinions	1-Not at all	2-Slightly	3-Somewhat	4-Moderately	5-Extremely	-
How effective do you think PrEP is in preventing individuals who take it every day as prescribed from getting HIV?	0 (0.0%)	2 (1.6%)	12 (9.8%)	26 (21.1%)	83 (67.5%)	4.54 (0.74)
How relevant do you think HIV prevention is amongst your clients?	1 (0.8%)	7 (5.7%)	17 (13.8%)	28 (22.8%)	70 (56.9%)	4.29 (0.96)
How important do you think it is for your clients to take PrEP to reduce their risk?	1 (0.8%)	5 (4.1%)	19 (15.5%)	30 (24.4%)	68 (55.3%)	4.29 (0.93)
Based on your understanding of PrEP side effects, how safe is PrEP?	0 (0.0%)	2 (1.6%)	16 (13.0%)	57 (46.3%)	48 (39.0%)	4.23 (0.73)

**PrEP Practicality**	**1-Strongly disagree**	**2-Somewhat disagree**	**3-Neither agree nor disagree**	**4-Somewhat agree**	**5-Strongly agree**	**-**
HIV prevention is important and relevant to the health of my clients.	9 (7.3%)	0 (0.0%)	6 (4.9%)	12 (9.8%)	96 (78.0%)	4.51 (1.11)
My patients have access to PrEP prescribing clinicians within my organization.	10 (8.1%)	1 (0.8%)	22 (17.9%)	16 (13.0%)	74 (60.2%)	4.16 (1.24)
My patients have access to PrEP prescribing clinicians outside my organization.	2 (1.6%)	4 (3.3%)	57 (46.3%)	22 (17.9%)	38 (30.9%)	3.73 (0.99)
I am concerned about PrEP side effects.	19 (15.5%)	21 (17.1%)	58 (47.2%)	21 (17.1%)	4 (3.3%)	2.76 (1.02)
Due to competing priorities, it is not practical to address HIV prevention with PrEP in routine encounters with my clients.	31 (25.2%)	19 (15.5%)	38 (30.9%)	26 (21.1%)	9 (7.3%)	2.70 (1.26)
Addressing HIV Prevention with PrEP is the role of other staff, not my role.	45 (36.6%)	12 (9.8%)	28 (22.8%)	21 (17.1%)	17 (13.8%)	2.62 (1.47)

**PrEP Confidence**	**1-Not at all confident**	**2-A little confident**	**3-Somewhat confident**	**4-Confident**	**5-Very confident**	**-**
I know where to refer my clients for PrEP.	7 (5.7%)	14 (11.4%)	13 (10.6%)	24 (19.5%)	65 (52.9%)	4.02 (1.27)
I am able to discuss PrEP with my clients.	21 (17.1%)	19 (15.5%)	23 (18.7%)	34 (27.6%)	26 (21.1%)	3.20 (1.39)
I have enough resources to give me clients regarding PrEP.	26 (21.1%)	17 (13.8%)	26 (21.1%)	34 (27.6%)	20 (16.3%)	3.04 (1.39)
My clients can adhere to PrEP.	11 (8.9%)	33 (26.8%)	56 (45.5%)	19 (15.5%)	4 (3.3%)	2.77 (0.93)
I know enough about current best practices for PrEP.	26 (21.1%)	29 (23.6%)	34 (27.6%)	16 (13.0%)	18 (14.6%)	2.76 (1.32)
I have the necessary skills and knowledge to assist my clients to take PrEP.	31 (25.2%)	25 (20.3%)	30 (24.4%)	18 (14.6%)	19 (15.5%)	2.75 (1.88)
My clients are motivated to take PrEP.	21 (17.1%)	37 (30.1%)	48 (39.0%)	14 (11.4%)	3 (2.4%)	2.52 (0.98)
My clients will have transportation to make their PrEP appointments.	25 (20.3%)	44 (35.8%)	32 (26.0%)	12 (9.8%)	10 (8.1%)	2.50 (1.16)
There is sufficient financial compensation for me in my role to address HIV prevention with PrEP with my clients.	46 (37.4%)	18 (14.6%)	35 (28.5%)	16 (13.0%)	8 (6.5%)	2.37 (1.28)
My clients can cover the costs associated with PrEP and PrEP care.	45 (36.6%)	31 (25.2%)	25 (20.3%)	14 (11.4%)	8 (6.5%)	2.26 (1.25)

**Fig. 1a:**
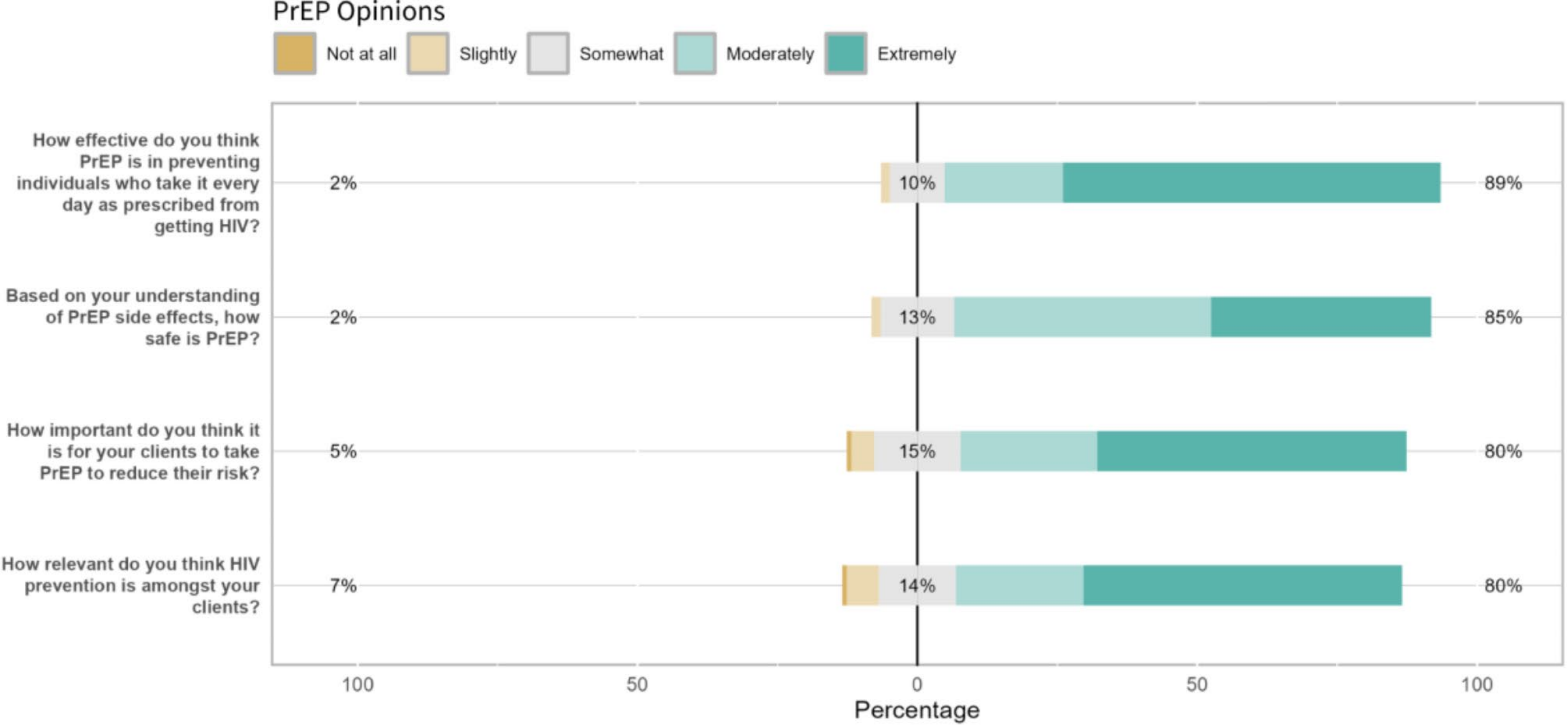
PrEP opinions among community-based staff (*n* = 123). The % statistics at each end of the X axes for Figs. 1a–1c represent cumulative positive or negative valence. For example, the left-aligned statistic is the proportion of respondents who either answered “1” or “2”; the middle statistic is the proportion of respondents who answered “3”; and the right-aligned statistic is the proportion of respondents who answered “4” or “5”. The percentages are rounded to the nearest whole number

**Fig. 1b:**
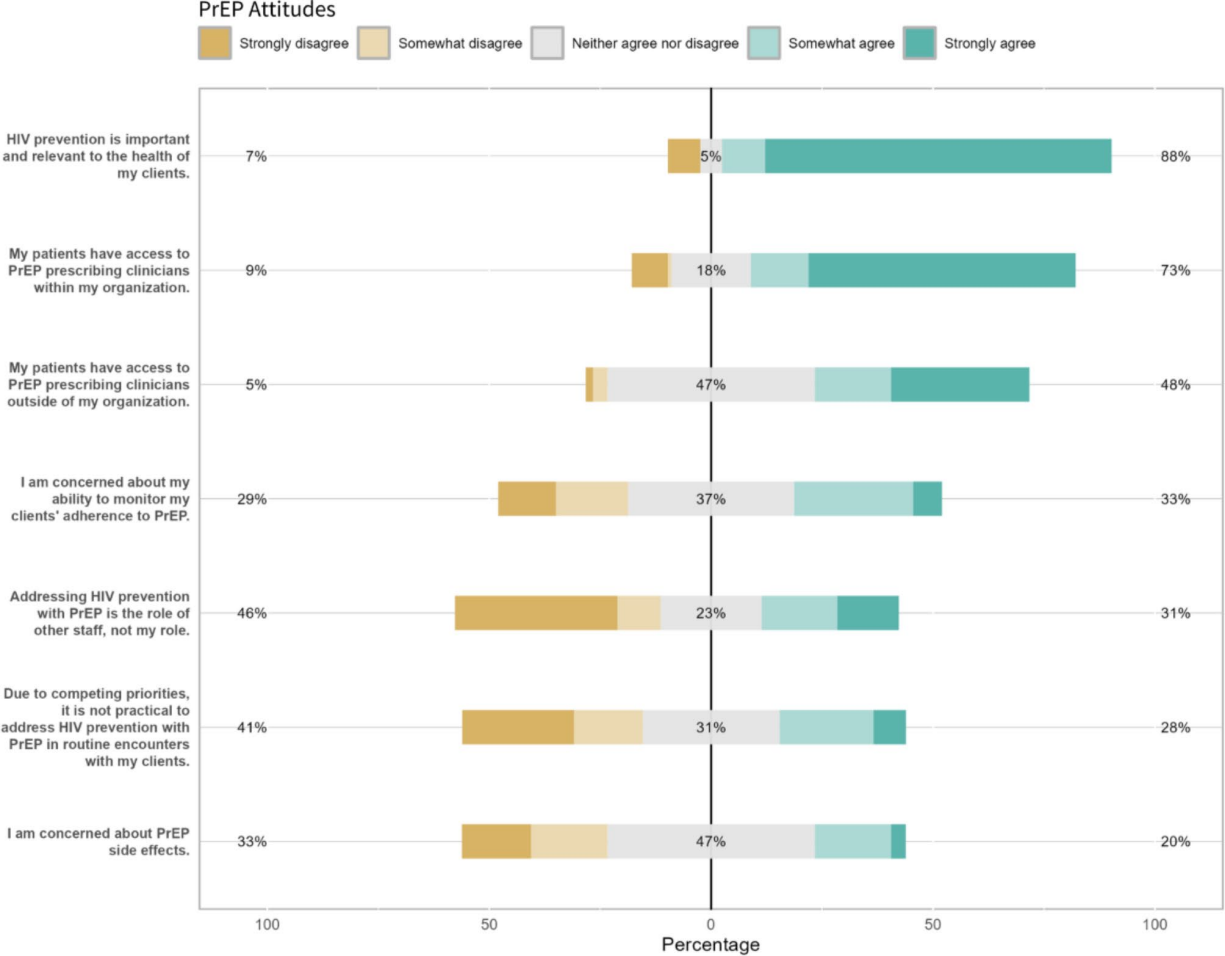
PrEP practicality attitudes among community-based staff (*n* = 123). The % statistics at each end of the X axes for Figs. 1a–1c represent cumulative positive or negative valence. For example, the left-aligned statistic is the proportion of respondents who either answered “1” or “2”; the middle statistic is the proportion of respondents who answered “3”; and the right-aligned statistic is the proportion of respondents who answered “4” or “5”. The percentages are rounded to the nearest whole number

**Fig. 1c:**
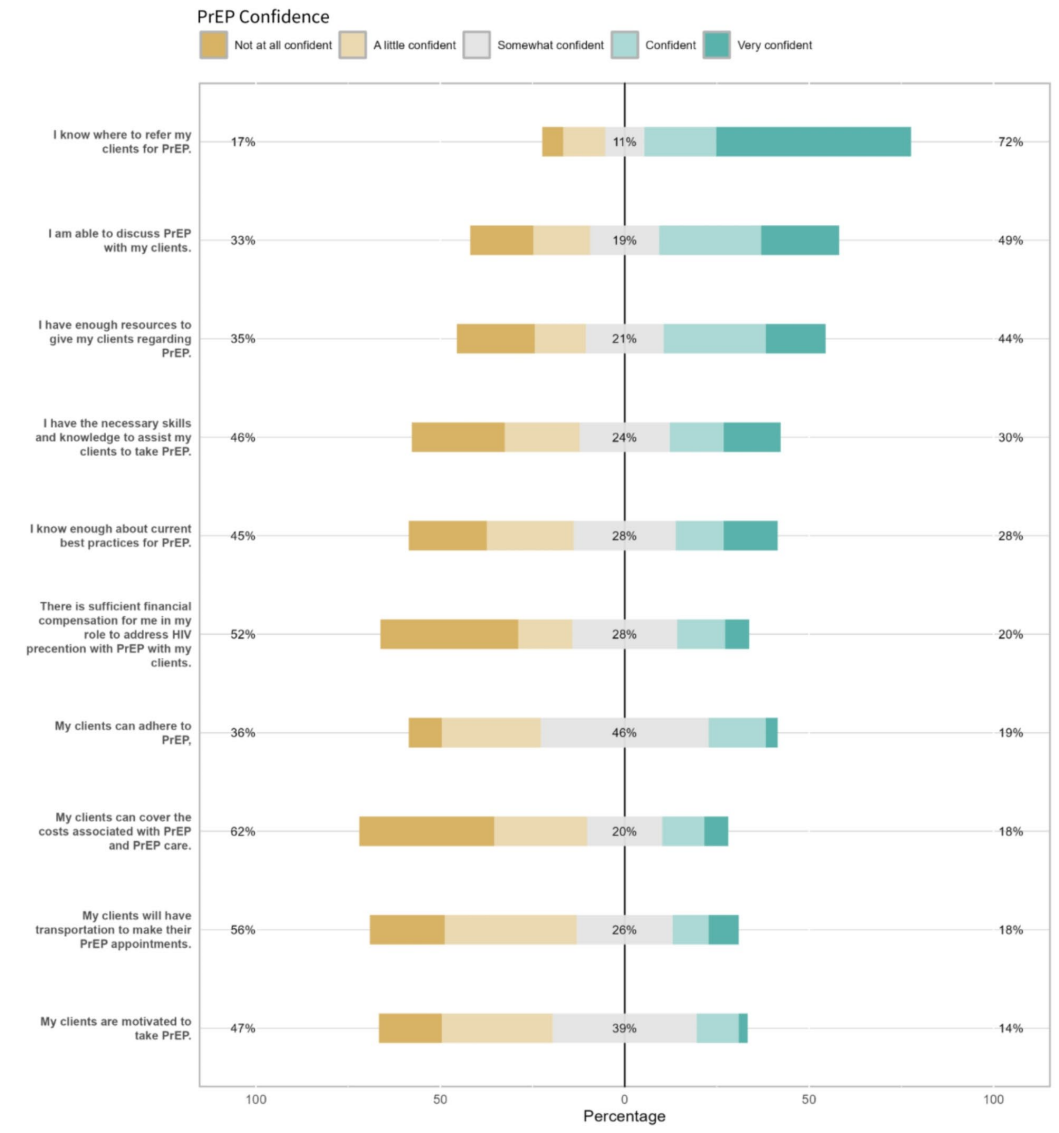
PrEP confidence among community-based staff (*n* = 123). The % statistics at each end of the X axes for Figs. 1a–1c represent cumulative positive or negative valence. For example, the left-aligned statistic is the proportion of respondents who either answered “1” or “2”; the middle statistic is the proportion of respondents who answered “3”; and the right-aligned statistic is the proportion of respondents who answered “4” or “5”. The percentages are rounded to the nearest whole number

### CMBQ overall responses

Respondents endorsed influential generalized (mean score = 2.1) and training-related (mean score = 2.5) implementation barriers less frequently than they indicated positive attitudes towards adopting CM (mean score = 3.6). Average responses to the individual items on the CMBQ and item-by-item response breakdowns are presented in Table 4.

**Table 4:** Contingency management beliefs questionnaire responses (*n* = 123)

	N (%) (*n* = 123)					Mean (SD)
Generalized Barriers Subscale	1-No influence at all	2-Very little influence	3-Some influence	4-Strong influence	5-Very strong influence	2.1 (0.7)
I think the research evidence about CM’s effectiveness does not apply to our everyday clients.	23 (18.7%)	15 (12.2%)	50 (40.7%)	22 (17.9%)	13 (10.6%)	2.9 (1.2)
I am worried about what happens once the contingencies are withdrawn.	25 (20.3%)	17 (13.8%)	51 (41.5%)	19 (15.5%)	11 (8.9%)	2.8 (1.2)
I am concerned clients might sell/trade earned items for drugs.	31 (25.2%)	26 (21.1%)	38 (30.9%)	20 (16.3%)	8 (6.5%)	2.6 (1.2)
CM doesn’t address the underlying cause of the clients’ health needs.	31 (25.2%)	28 (22.8%)	40 (32.5%)	15 (12.2%)	9 (7.3%)	2.5 (1.2)
CM might cause arguments among clients (e.g., when some get prizes and other do not).	39 (31.7%)	29 (23.6%)	36 (29.3%)	11 (8.9%)	8 (6.5%)	2.3 (1.2)
I think that providing prizes undermines the clients’ internal motivation to reduce opioid use.	45 (36.6%)	28 (22.8%)	36 (29.3%)	5 (4.1%)	9 (7.3%)	2.2 (1.2)
I do not have time to administer prizes in my routine sessions.	56 (45.5%)	25 (20.3%)	29 (23.6%)	4 (3.3%)	9 (7.3%)	2.1 (1.2)
My clinical experience with individuals with substance use is more important than any research evidence.	52 (42.3%)	25 (20.3%)	34 (27.6%)	5 (4.1%)	7 (5.7%)	2.1 (1.2)
The community wouldn’t understand (i.e., clinic will look bad for giving rewards to individuals who use opioids).	49 (39.8%)	34 (27.6%)	28 (22.8%)	8 (6.5%)	4 (3.3%)	2.1 (1.1)
CM is expensive (e.g., cost of prizes).	53 (43.1%)	28 (22.8%)	34 (27.6%)	3 (2.4%)	5 (4.1%)	2.0 (1.1)
It seems like CM interventions create extra work for me.	55 (44.7%)	29 (23.6%)	30 (24.4%)	5 (4.1%)	4 (3.3%)	2.0 (1.1)
A lot of my clients are already abstinent from opioids at intake, so they don’t need CM.	52 (42.3%)	35 (28.5%)	28 (22.8%)	5 (4.1%)	3 (2.4%)	2.0 (1.0)
I think clients will view CM as patronizing.	52 (42.3%)	35 (28.5%)	27 (22.0%)	6 (4.9%)	3 (2.4%)	2.0 (1.0)
I am not convinced by the research about CM’s effectiveness.	65 (52.9%)	19 (15.5%)	29 (23.6%)	7 (5.7%)	3 (2.4%)	1.9 (1.1)
I believe it is not right to give rewards for abstinence from opioids if clients are not meeting other treatment goals (e.g., PrEP adherence).	64 (52.0%)	25 (20.3%)	26 (21.1%)	1 (0.8%)	7 (5.7%)	1.9 (1.1)
Our clinic rules prevent urine screening for opioid use.	82 (66.7%)	11 (8.9%)	17 (13.8%)	2 (2.4%)	10 (8.1%)	1.8 (1.3)
I find CM distasteful because it is basically paying someone to do what they should do already.	74 (60.2%)	22 (17.8%)	19 (15.5%)	5 (4.1%)	3 (2.4%)	1.7 (1.0)

**Training-related Barriers Subscale**	**1-No influence at all**	**2-Very little influence**	**3-Some influence**	**4-Strong influence**	**5-Very strong influence**	**2.5 (0.9)**
I want more training before implementing CM.	20 (16.3%)	14 (11.4%)	44 (35.8%)	22 (17.9%)	23 (18.7%)	3.1 (1.3)
I don’t feel qualified or properly trained to administer CM interventions.	40 (32.5%)	17 (13.8%)	42 (34.2%)	12 (9.8%)	12 (9.8%)	2.5 (1.3)
Currently, no one in my facility has the experience to supervise CM.	53 (43.1%)	18 (14.6%)	36 (29.3%)	7 (5.7%)	9 (7.3%)	2.2 (1.3)
My agency / supervisors / administrators do not support CM (e.g., do not provide training, resources).	60 (48.8%)	17 (13.8%)	34 (27.5%)	6 (4.9%)	6 (4.9%)	2.0 (1.2)

**Pro-Contingency Management Items Subscale**	**1-No influence at all**	**2-Very little influence**	**3-Some influence**	**4-Strong influence**	**5-Very strong influence**	**3.6 (0.8)**
Any source of motivation, including extrinsic motivation, is good if it helps get clients involved and responding to treatment.	5 (4.1%)	6 (4.9%)	32 (26.0%)	37 (30.1%)	43 (35.0%)	3.9 (1.1)
I think that CM is worth the time and effort if it works.	6 (4.9%)	7 (5.7%)	31 (25.2%)	39 (31.7%)	40 (32.5%)	3.8 (1.1)
I am in favor of adding CM interventions to our existing services.	5 (4.1%)	10 (8.1%)	33 (26.8%)	35 (28.5%)	40 (32.5%)	3.8 (1.1)
CM is useful when targeting treatment goals for opioid use disorder other than abstinence from opioids (attendance, activities).	2 (1.6%)	7 (5.7%)	48 (39.0%)	40 (32.5%)	26 (21.1%)	3.7 (0.9)
CM is helpful because it helps keep clients engaged in treatment long enough for them to really learn valuable skills.	6 (4.9%)	5 (4.1%)	47 (38.2%)	35 (28.5%)	30 (24.4%)	3.6 (1.1)
It seems to me that CM is good for clients because they get excited about their treatment and progress.	6 (4.9%)	4 (3.3%)	46 (37.4%)	43 (35.0%)	24 (19.5%)	3.6 (1.0)
I think CM focuses on the good in clients’ behavior, and not just what went wrong.	10 (8.1%)	7 (5.7%)	44 (35.8%)	31 (25.2%)	31 (25.2%)	3.5 (1.2)
I think CM will help get clients in the door (e.g., motivate them to come to treatment).	6 (4.9%)	10 (8.1%)	47 (38.2%)	33 (26.8%)	27 (22.0%)	3.5 (1.1)
CM is useful when targeting opioid abstinence.	5 (4.1%)	10 (8.1%)	47 (38.2%)	35 (28.5%)	26 (21.1%)	3.5 (1.0)
CM helps clients reduce their opioid use so that they can work on other aspects of treatment.	8 (6.5%)	10 (8.1%)	52 (42.5%)	29 (23.6%)	24 (19.5%)	3.4 (1.1)
CM is good for the client-counselor relationship.	10 (8.1%)	13 (10.6%)	52 (42.3%)	30 (24.4%)	18 (14.6%)	3.3 (1.1)

**Additional Items**	**1-No influence at all**	**2-Very little influence**	**3-Some influence**	**4-Strong influence**	**5-Very strong influence**	-
It is preferable to give clients prizes in choice of goods/supplies/gift cards (rather than cash) for reaching treatment goals.	9 (7.3%)	8 (6.5%)	32 (26.0%)	43 (27.6%)	40 (32.5%)	3.7 (1.2)
CM is useful for targeting HIV prevention with PrEP.	3 (2.4%)	5 (4.1%)	50 (40.7%)	38 (30.9%)	27 (22.0%)	3.7 (0.9)
It is okay for a client to have the opportunity to earn prizes worth as much as $100 for reaching treatment goals.	12 (9.8%)	11 (8.9%)	33 (26.8%)	34 (27.6%)	33 (26.8%)	3.5 (1.3)
The activity contracting in CM allows us to individualize goals to a specific client’s needs.	15 (12.2%)	14 (11.4%)	42 (34.2%)	37 (30.1%)	15 (12.2%)	3.2 (1.2)
Urine testing is easy to fit into my workflow.	23 (18.7%)	16 (13.0%)	40 (32.5%)	21 (17.1%)	23 (18.7%)	3.0 (1.3)
Reinforcing PrEP adherence via urine testing will help motivate clients to be consistent with their medication.	14 (11.4%)	22 (17.9%)	52 (42.9%)	21 (17.1%)	14 (11.4%)	3.0 (1.1)
Because many of our clients are difficult to contact regularly, CM is not feasible.	32 (26.0%)	26 (21.1%)	52 (42.3%)	8 (6.5%)	5 (4.1%)	2.4 (1.1)
It is preferable to give clients prizes in cash for reaching treatment goals.	48 (39.0%)	21 (17.1%)	31 (25.2%)	12 (9.8%)	11 (8.9%)	2.3 (1.3)
CM is not flexible enough for our clients who may not be ready to make changes.	44 (35.8%)	25 (20.3%)	40 (32.5%)	9 (7.3%)	5 (4.1%)	2.2 (1.1)
I feel like CM targeting opioid abstinence is not compatible with a harm reduction approach.	53 (43.1%)	25 (20.3%)	32 (26.0%)	8 (6.5%)	5 (4.1%)	2.1 (1.1)
It seems like activity contracting takes too much time.	45 (36.6%)	32 (26.0%)	35 (28.5%)	7 (5.7%)	4 (3.3%)	2.1 (1.1)
Finding verifiable activities for CM is too difficult and time-consuming.	47 (38.2%)	26 (21.1%)	39 (31.7%)	7 (5.7%)	4 (3.3%)	2.1 (1.1)
Our clients will not be interested in prizes for opioid abstinence.	60 (48.8%)	27 (22.0%)	22 (17.9%)	7 (5.7%)	7 (5.7%)	2.0 (1.2)
Our clients will not be interested in prizes for PrEP adherence.	63 (51.2%)	22 (17.9%)	23 (18.7%)	8 (6.5%)	7 (5.7%)	2.0 (1.2)
I believe it is not right to give rewards for PrEP if clients are not meeting other treatment goals (e.g., MOUD engagement).**	60 (49.2%)	29 (23.8%)	25 (20.5%)	3 (2.5%)	5 (4.1%)	1.9 (1.1)

**NA = 1. CM = contingency management

### Generalized barriers

The generalized barriers with the highest rated influence on implementing CM included: “I think the research evidence about contingency management’s effectiveness does not apply to our everyday clients.” (mean score = 2.9), followed by “I am worried about what happens once the contingencies are withdrawn.” (mean score = 2.8), and “I am concerned clients might sell/trade earned items for drugs.” (mean score = 2.6). The least commonly endorsed barriers included: “I find contingency management distasteful because it is basically paying someone to do what they should do already.” (mean score = 1.7) and “Our clinic rules prevent urine screening for opioid use.” (mean score = 1.8).

### Training-related barriers

Looking at training-related barriers, the most endorsed included: “I want more training before implementing contingency management.” (mean score = 3.1) and “I don’t feel qualified or properly trained to administer contingency management interventions.” (mean score = 2.5). The least commonly endorsed training-related barriers were: “My agency / supervisors / administrators do not support contingency management (e.g., do not provide training, resources).” (mean score = 2.0) and “Currently, no one in my facility has the experience to supervise contingency management.” (mean score = 2.2).

### Support for CM

The highest rated items indicating support for CM included: “Any source of motivation, including extrinsic motivation, is good if it helps get clients involved and responding to treatment.” (mean score = 3.9), “I think that contingency management is worth the time and effort if it works.” (mean score = 3.8), and “I am in favor of adding contingency management interventions to our existing services.” (mean score = 3.8). The lowest rated items were: “Contingency management is good for the client-counselor relationship.” (mean score = 3.3) and “Contingency management helps clients reduce their opioid use so that they can work on other aspects of treatment.” (mean score = 3.4).

### Contingency management to promote PrEP

When reviewing additional items related to the use of CM to promote PrEP initiation and adherence, the highest endorsed items were: “Contingency management is useful for targeting HIV prevention with PrEP.” (mean score = 3.7) and “It is preferable to give clients prizes in choice of goods/supplies/gift cards (rather than cash) for reaching treatment goals.” (mean score = 3.7). Items indicating the lowest influence for implementing CM for HIV prevention included: “I believe it is not right to give rewards for PrEP if clients are not meeting other treatment goals.” (mean score = 1.9); “Our clients will not be interested in prizes for opioid abstinence.” (mean score = 2.0); and “Our clients will not be interested in prizes for PrEP adherence.” (mean score = 2.0).

## Discussion

These findings suggest high feasibility and acceptability related to the use of CM to promote PrEP in various service provision settings. Participants generally rated items indicating positive attitudes towards CM more highly than they rated items related to barriers to implementing CM. Compared to other recent studies using the CMBQ, this study observed lower ratings for both generalized and training-related barriers and equal or higher ratings for supportive statements [35, 36]. These scores could be further improved through training and education of organization staff on CM [37].

The most commonly endorsed barriers to CM related to the need for more training on the evidence behind CM and how to implement the components of CM within different settings. Participant responses also indicated a level of concern around how CM participants might use the prizes they earn. These concerns are similar to those observed in other research assessing implementation barriers related to CM [38].

With regard to PrEP itself, participants expressed positive attitudes, strongly endorsing its relevance, importance, efficacy and safety. This finding represents an important departure from the extant literature documenting provider and other non-clinical staff concerns about PrEP [39–41], which ultimately interfere with adoption [22]. Despite the favorable regard for PrEP amongst participants, their overall lack of confidence in best practices and discussing PrEP initiation with clients corroborates findings from past studies of provider awareness and comfortability [42–44]. This self-reported competency gap, in addition to sustained concerns about client capacity for PrEP (i.e., motivation, adherence, cost), may be notable barriers to the implementation of both PrEP in general as well as CM to support PrEP among PWID (4, 45–46). Trainings around CM for PrEP adherence should directly address CM’s long and robust history of promoting new behavior initiation and adherence over time, as well as highlighting the suitability of PrEP adherence as a behavioral target in CM protocols. For example, CM requires objective and verifiable target behaviors. PrEP offers multiple options of adherence verification, such as direct observation for injections or video verification or urine testing for patients taking daily oral formulations.

### Limitations

These findings should be taken into consideration along with the limitations of the study. All participants were from the northeastern US, based at organizations participating in a clinical trial of a CM intervention. This sample may not be generalizable to other settings. Additionally, given data were collected during the COVID-19 pandemic, responses may differ based on how respondent priorities changed. Finally, we did not calculate associations between CMBQ scores and other variables due to limited power.

## Conclusions

Overall, respondents understood the value of CM in motivating clients and thought it would support HIV prevention activities, including PrEP engagement. Positive attitudes towards PrEP signaled increased potential for readiness to implement this intervention with an underserved population. These results suggest staff are favorable towards the use of CM in community-based organizations, though staff competency and concerns about providing PrEP-related care must also be considered. Continued efforts to research and increase utilization of CM in promoting various health behaviors across various settings is needed.

## Data Availability

The datasets used and/or analyzed during the current study are available from the corresponding author on reasonable request.

## Abbreviations

CM: Contingency management
CMBQ: Contingency Management Beliefs Questionnaire
MOUD: Medications for Opioid Use Disorder
PrEP: Pre–Exposure Prophylaxis for HIV Prevention
PWID: People Who Inject Drugs
